# A hybrid DDA/DIA-PASEF based assay library for a deep proteotyping of triple-negative breast cancer

**DOI:** 10.1038/s41597-024-03632-2

**Published:** 2024-07-18

**Authors:** Petr Lapcik, Klara Synkova, Lucia Janacova, Pavla Bouchalova, David Potesil, Rudolf Nenutil, Pavel Bouchal

**Affiliations:** 1 Department of Biochemistry, Faculty of Science, Masaryk University, Brno, Czech Republic; 2 Central European Institute of Technology, Masaryk University, Brno, Czech Republic; 3 Department of Oncological Pathology, Masaryk Memorial Cancer Institute, Brno, Czech Republic

**Keywords:** Biochemical networks, Predictive markers

## Abstract

Triple-negative breast cancer (TNBC) is the most aggressive subtype of breast cancer, and deeper proteome coverage is needed for its molecular characterization. We present comprehensive library of targeted mass spectrometry assays specific for TNBC and demonstrate its applicability. Proteins were extracted from 105 TNBC tissues and digested. Aliquots were pooled, fractionated using hydrophilic chromatography and analyzed by LC-MS/MS in data-dependent acquisition (DDA) parallel accumulation-serial fragmentation (PASEF) mode on timsTOF Pro LC-MS system. 16 individual lysates were analyzed in data-independent acquisition (DIA)-PASEF mode. Hybrid library was generated in Spectronaut software and covers 244,464 precursors, 168,006 peptides and 11,564 protein groups (FDR = 1%). Application of our library for pilot quantitative analysis of 16 tissues increased identification numbers in Spectronaut 18.5 and DIA-NN 1.8.1 software compared to library-free setting, with Spectronaut achieving the best results represented by 190,310 precursors, 140,566 peptides, and 10,463 protein groups. In conclusion, we introduce assay library that offers the deepest coverage of TNBC proteome to date. The TNBC library is available via PRIDE repository (PXD047793).

## Background & Summary

Triple-negative breast cancer (TNBC) is an aggressive disease that represents about 15% of all breast cancer cases and is typically associated with a poor prognosis^1,2^. Approximately 28–39% of the TNBC patients do not survive 5 years after the diagnosis, leading to shorter survival rates compared to other breast tumor subtypes^3^. TNBC tumors are defined by the lack of expression of estrogen receptor, progesterone receptor and human epidermal growth factor receptor-2 (HER2)^4^. The absence of these receptors that are targets of biological therapy for other breast cancer subtypes results in insensitivity of TNBC tumors to hormonal and anti-HER2 therapies, leading to untargeted cytotoxic chemotherapy as a mainstay systemic treatment of TNBC tumors^5,6^. Moreover, TNBC tumors exhibit a significant molecular heterogeneity^7^ that complicates development of targeted therapies for TNBC patients. A deep proteome coverage of TNBC tumors is essential for development of precise classification of TNBC tumors and identification of novel therapeutic targets.

Mass spectrometry-based techniques allow large-scale identification and quantification of proteins and show potential for addressing clinical issues^8^. The data-independent acquisition (DIA) strategies offer accurate and reproducible protein quantification across large groups of samples with reduced missing value numbers^9,10^, as demonstrated also for breast cancer tissues^11^. To assign the elution groups of fragment ions to precursors and peptides, analysis of DIA data requires a spectral library. Typically, a library-based approach is being used, and the spectral library can be generated from data-dependent acquisition (DDA) LC-MS/MS measurements of individual samples or their fractionated pools^12,13^. As an alternative, an approach that implements libraries predicted *in silico* from protein sequence databases or constructed from DIA data as pseudospectra have been employed^14,15^.

Furthermore, the use of sample type-specific spectral libraries is beneficial for precise peptide and protein identification^16^ and can offer better proteome coverage compared to pan-human libraries^17^. García-Adrián *et al*. recently published a DIA dataset including 125 TNBC tumors measured on LTQ-Orbitrap Fusion Lumos mass spectrometer that covers 3092 proteins, of which 1206 proteins were consistently detected and quantified in at least 66% of the samples^18^. The application of this dataset for definition of TNBC subgroups and novel therapeutic targets is however limited by the low number of protein identifications. As novel mass spectrometry instrumentation and strategies have been developed, there is potential for generation of more comprehensive TNBC-specific DIA datasets and spectral libraries. Particularly four-dimensional proteomics technology implementing trapped ion mobility spectrometry significantly improved sensitivity of peptide detection^19^. This technology implemented in parallel accumulation-serial fragmentation (PASEF) strategy allows multiplication of the sequencing speed without any loss of sensitivity^20^. PASEF can be performed in a DIA mode (DIA-PASEF) that utilizes smaller number of ions in particular DIA windows and improves specificity, offers near 100% ion utilization and high degree of reproducibility as well as quantitative accuracy^21^. Moreover, several software strategies have been developed for DIA data extraction from the raw runs^22^, from which, Spectronaut^23^ and DIA-NN^24^ represent two popular tools that are also capable of processing the DIA-PASEF data^25,26^. Spectronaut implements database search engine Pulsar that allows targeted DIA data extraction using spectral libraries generated from DDA runs. Moreover, Spectronaut incorporates library-free search strategy called directDIA for targeted data extraction after a direct search of DIA data by generating pseudo MS/MS spectra^14^. Combination of these approaches allows generation of hybrid libraries^27^ that combine searches of both DDA and DIA data using Pulsar search engine, resulting in a deep proteome coverage and improved retention time calibration. On the other hand, DIA-NN generates libraries by prediction from protein sequence databases for library-free searches, nevertheless it allows targeted DIA data extraction using spectral libraries generated in external tools. DIA-NN software offers a fast performance which is valuable for high-throughput applications^24^.

**Fig. 1:**
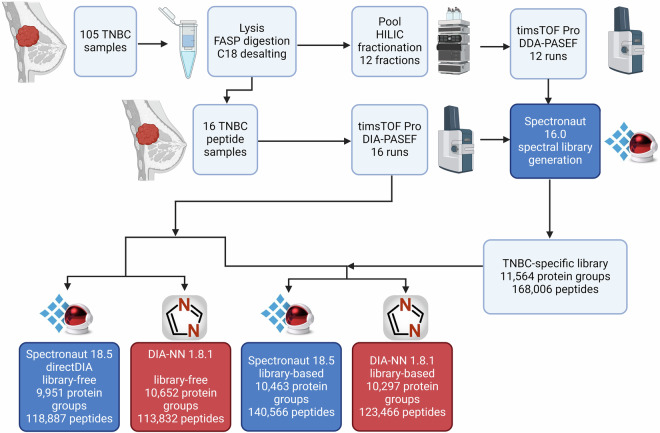
Workflow of the study. Generation of TNBC-specific targeted proteomics assay library using data from fractions of the pool of 105 TNBC tissues and 16 individual samples and quantitative data extraction in Spectronaut and DIA-NN in library-based and library-free settings. The figure was created with BioRender.com.

To generate a comprehensive proteomics assay library specific for TNBC, we fractionated peptides from a large set of TNBC tissues, analyzed the fractions using timsTOF Pro LC-MS system in DDA-PASEF mode and 16 of the individual samples in DIA-PASEF mode, generated a new hybrid library of targeted proteomics assays in Spectronaut software, and compared the library-based and library-free DIA data extraction strategies using two key software tools, Spectronaut and DIA-NN (Fig. 1).

## Methods

### Sample information and patient characteristics

Aliquots of primary tumor tissue lysates from 105 patients treated at Masaryk Memorial Cancer Institute (MMCI) in Brno, Czech Republic, were used to generate the spectral library. Only samples from women diagnosed with a breast cancer with negative status of estrogen, progesterone and human epidermal growth factor receptors using immunohistochemistry, who were treated at MMCI between 2001–2015 and did not receive any neoadjuvant treatment before the tumor resection, were included into the study. The study was approved by Ethics committees of MMCI (2019/1543/MOU, 2021/1723/MOU) and Masaryk University (EKV-2021-083). All patients have signed the informed consent. For the patient characteristics please see Supplementary File 1.

### Sample preparation

The tissue samples were received in 20 min after the surgical removal, evaluated by a pathologist and snap frozen in liquid nitrogen. The pieces of tumor tissue corresponding to 2 × 2 × 2 mm blocks were subjected to lysis in 200 µL of 6 M guanidine hydrochloride and 1% Triton X-100 in 0.1 M phosphate buffer pH 6.6. The tissue samples were subsequently homogenized in the homogenizer (Retsch, Haan, Germany) for 2 × 2 min at 25 s^−1^. The samples were sonicated 30 × 0.1 s, power 50 W with a needle sonicator (Bandelin HD 2200; Bandelin, Berlin, Germany). The protein lysates were next incubated for 60 min at RT and centrifuged at 14,000 g/20 min/4 °C. The protein concentrations in the supernatants were measured using the RC-DC protein assay (Bio-Rad, Hercules, CA, USA). The protein samples were subjected to trypsin digestion with the use of Filter-Aided Sample Preparation (FASP) method^28^ with modifications. 100 µg of protein per sample was transferred to the Microcon filter device, cut-off 30 kDa (Millipore) with 100 µL of 8 M urea in 0.5 triethylammonium bicarbonate (TEAB) pH 8.5 and centrifuged at 14,000 g/15 min. The samples were reduced with 10 µL of 50 mM tris(2-carboxyethyl)phosphine) (Sigma-Aldrich) in 100 µL of 8 M urea in 0.5 M TEAB pH 8.5 for 60 min/600 rpm and centrifuged at 14,000 g/15 min. Next, the samples were alkylated using 5 µL of 200 mM S-methyl methanethiosulfonate (Sigma-Aldrich) in 100 µL of 8 M urea in 0.5 M TEAB pH 8.5 for 1 min/600 rpm and for 20 min in the dark without shaking, and centrifuged at 14,000 g/15 min. The protein samples on the filter were washed twice with 100 µL of 0.5 M TEAB pH 8.5 and centrifuged at 14,000 g/20 min. The proteins were digested by 3.33 µL of trypsin (Promega, 1 µg/µL) in the trypsin:protein ratio 1:30 in 100 µL of 0.5 M TEAB pH 8.5 for 16 hrs at 37 °C. Resulting peptides were eluted with centrifugation at 14,000 g/15 min and desalted on MicroSpin columns C18 (Nest Group) as described in^29^ with modifications. First, the columns were washed twice with 200 µL of 0.1% TFA in 100% acetonitrile (ACN) and centrifuged at 100 g/3 min. The columns were washed with 200 µL of 0.1% TFA in water and centrifuged at 300 g/3 min. Another 200 µL of 0.1% TFA in water was added to the columns followed by 15 min incubation and centrifugation at 300 g/3 min. The peptide samples were then added to the hydrated columns, centrifuged at 500 g/3 min, and washed three times with addition of 200 µL of 0.1% TFA in water and centrifugation at 500 g/ 3 min. The desalted peptides were eluted in three steps with addition of 200 µL 0.1% TFA in 50% ACN, 200 µL of 0.1% TFA in 80% ACN, and 200 µL of 0.1% TFA in 100% ACN, with centrifugation at 500 g/3 min between each step. The eluted peptides were vacuum-dried in a SpeecVac concentrator and stored at −20 °C.

### Peptide fractionation

For the spectral library generation, 90 µL aliquots of desalted peptide eluates corresponding to 15 µg of starting protein material were transferred from individual samples and pooled. 800 µg of the pooled sample was subjected to hydrophilic chromatography (HILIC) fractionation as described previously^28^ with the use of HILIC Kinetex column (Phenomenex, USA, 2.6 μm, 150 × 2.1 mm, 100 A) connected to Agilent Infinity 1260 LC system (Agilent, USA) with mobile phase (A) 50 mM ammonium formate pH 3.2 in 100% acetonitrile, and mobile phase (B) of 50 mM ammonium formate pH 3.2 in water. The fractionation was performed with isocratic 5% B for 5 min, 7 min gradient to 25% B, then 23 min gradient to 39% B followed by 5 min gradient to 55% B, isocratic 55% B for 5 min, 0.5 min gradient to 5% B and subsequently isocratic 5% B for 4.5 min. Fractions were collected every 1 min, neighboring fractions with lower signal intensities were subsequently pooled to generate a final set of 12 fractions based on peptide content (Supplementary Fig. S1). The peptides were vacuum-dried in a SpeedVac concentrator and stored at −20 °C.

### LC-MS/MS analysis

All peptide mixtures were extracted into LC-MS vials by 2.5% formic acid (FA) in 50% ACN and 100% ACN with the addition of polyethylene glycol (final concentration 0.001%)^30^ and concentrated in a SpeedVac concentrator (Thermo Fisher Scientific) and peptide concentration was estimated using small aliquot measurement using UltiMate 3000 RSLCnano system (Thermo Fisher Scientific) online connected to Impact II system (Bruker) using the area under the total ion chromatogram curve as a total peptide quantity estimate and using external calibration curve done using HeLa (Pierce) solutions. LC-MS/MS analyses of all peptide extracts (200 ng per injection based on the estimated total peptide concentration) from 12 HILIC fractions and 16 individual samples from the original sample set used for generation of the pooled sample for HILIC fractionation were performed using nanoElute system (Bruker) directly connected to timsTOF Pro mass spectrometer (Bruker) as described previously^31^ with modifications for DDA-PASEF measurements.

MSn data were acquired in DDA-PASEF for the HILIC fractions and DIA-PASEF approach for the individual samples. m/z range of 100–1700 and 1/k0 range of 0.6–1.6 V × s × cm^−2^ was used in both methods. DDA-PASEF method acquired 10 PASEF scans with scheduled target intensity of 20,000 and intensity threshold of 2,500. Active exclusion was set for 0.4 min with precursor reconsideration for 4 × more intense precursors. DIA-PASEF method used m/z 400–1100 precursor range and equal windows size of 26 Th (incl. 1 Th overlaps) using two steps each PASEF scan and cycle time of 100 ms locked to 100% duty cycle (Supplementary File 2).

### DDA data processing and spectral library generation

The DDA data from 12 HILIC peptide fractions and 16 DIA runs of individual samples used for spectral library generation were searched by Pulsar algorithm implemented in Spectronaut 16.0 software (Biognosys) against human UniProt/SwissProt database (version 2022_03 downloaded on 2022-09-23, 20,398 sequences). Enzyme specificity was set to trypsin/P, two missed cleavages were allowed, fixed modifications were set to methylthiolation (C), and variable modifications were set to oxidation (M) and acetylation (protein N-terminus). Protein, peptide and peptide-spectrum match (PSM) FDR were set to 0.01. MS1 and MS2 tolerances were set as dynamic with correction factor 1. Other parameters were set as default.

### DIA data processing in Spectronaut software

The 16 TNBC samples measured in DIA-PASEF mode used for library generation were subjected to quantitative data extraction in Spectronaut 18.5 software using our hybrid library generated in Spectronaut 16.0. Precursor Qvalue cutoff and experiment protein Qvalue cutoff were set to 0.01. No missing value imputation was used. Other parameters were set as default. The results were reported from sparse profiles including peptides identified in at least one sample with Qvalue < 0.01^32^.

### Statistical analysis

The protein profile boxplots, distribution of missing values and data completeness were visualized using DIA-Analyst tool^33^. The plot of protein level coefficient of variation (CV) distribution was generated with MixProTool^34^.

## Data Records

The raw DDA-PASEF mass spectrometry proteomics data of peptide fraction measurements, Pulsar spectral library files, raw DIA-PASEF data for 16 samples used for targeted data extraction as well as Spectronaut and DIA-NN data analysis outputs have been deposited in the ProteomeXchange Consortium via the Proteomics Identifications (PRIDE) partner repository^35^ (http://www.ebi.ac.uk/pride/archive/) with the dataset identifier PXD047793^36^.

## Technical Validation

**Fig. 2:**
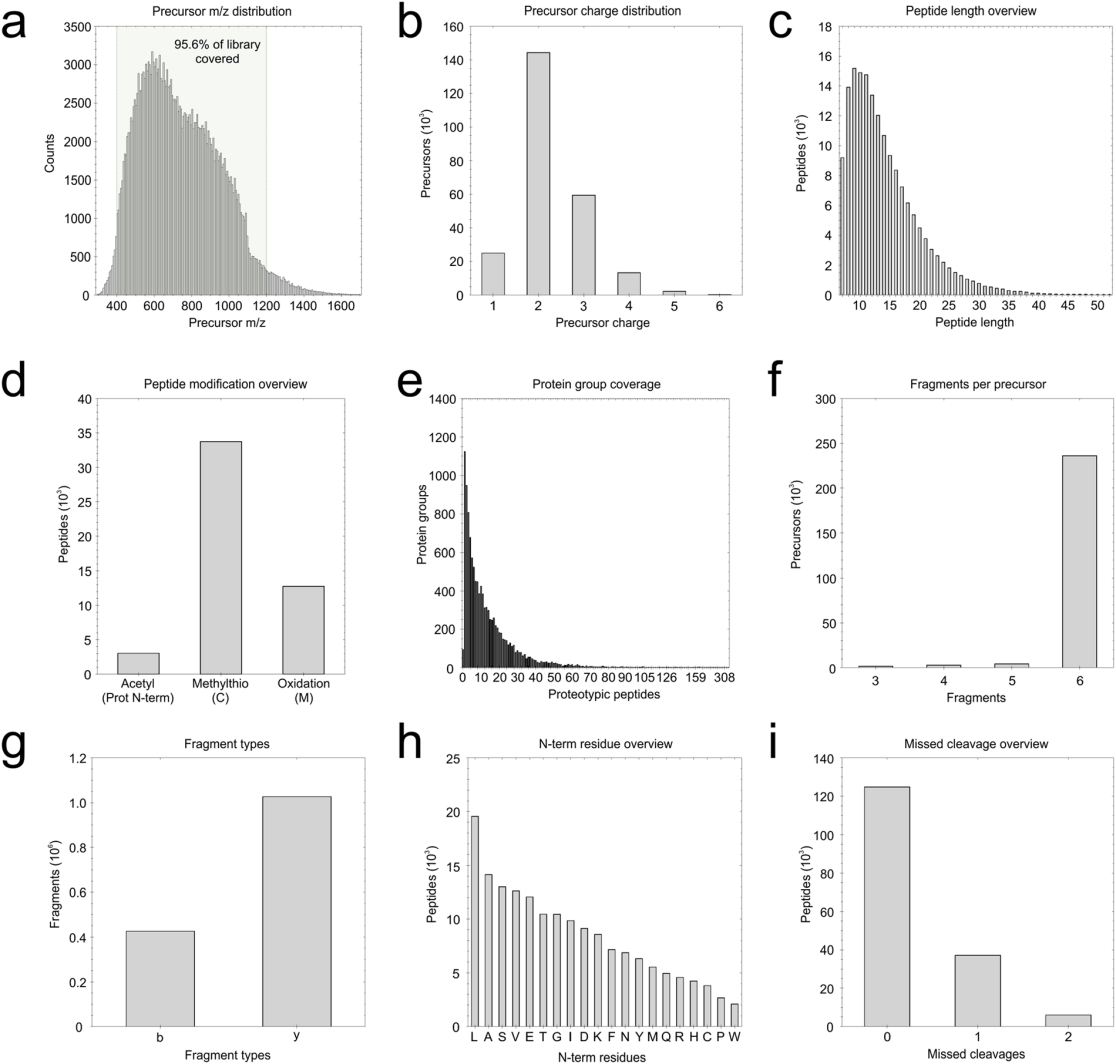
Characteristics of the TNBC-specific spectral library. (**a**) m/z scale of the precursors. (**b**) Precursor charge distribution. (**c**) Peptide length distribution. (**d**) Numbers of identified modified peptides. (**e**) Distribution of numbers of proteotypic peptides that were identified per protein. (**f**) Numbers of fragments per precursor. (**g**) Fragment ion types. (**h**) N-terminal amino acid distribution. (**i**) Missed cleavage rate.

For generation of TNBC-specific library of proteomics assays, peptides from total of 105 TNBC tissues were pooled and fractionated by HILIC (Supplementary Fig. S1) and subsequently conducted to LC-MS/MS analysis on timsTOF Pro mass spectrometer in DDA-PASEF mode, whereas 16 of the individual peptide samples from the original sample group used for HILIC fractionation were measured in DIA-PASEF mode (Supplementary File 2). Spectronaut 16.0 (Biognosys) software was used to build a hybrid library of assays consisting of a Pulsar search archive (*.psar file) of 16 DIA-PASEF measurements of individual samples, and a Pulsar search archive with 12 DDA-PASEF measurements of HILIC fractions and is accessible via PRIDE together with the raw data files as well as processed data files (Supplementary File 3). This final library includes 244,464 precursors and 168,006 stripped peptide sequences corresponding to 11,564 protein groups (FDR = 0.01). For 95.6% of precursors, the range of the mass was between 400 and 1,200 m/z (Fig. 2a) with one (10.2%), two (59.0%), three (24.3%) and four (5.4%) positive charges (Fig. 2b). The peptide length varied from 7 to 52 amino acids (Fig. 2c). The modifications included oxidation on methionine for 12,777 peptides, acetylation on N-terminus for 3,055 peptides, and methylthiolation on cysteine for 33,749 peptides (Fig. 2d). Of these, minimum of two proteotypic peptides were identified for 10,347 protein groups, and at least eight proteotypic peptides were identified for 6,375 protein groups (Fig. 2e). Precursors had mostly (235,979 precursors) more than 5 fragment ions (Fig. 2f), with the most prevalent (70.7%) y-ion series (Fig. 2g). The most frequent N-terminal amino acid was leucine (19,554 peptides) (Fig. 2h) and the protein digestion efficacy was demonstrated by 124,827 peptides with no missed cleavage (Fig. 2i).

## Usage Notes

The goal of this work is the generation of the comprehensive library of assays specific for TNBC in order to support DIA proteomics research of TNBC allowing deep proteome coverage.

**Fig. 3:**
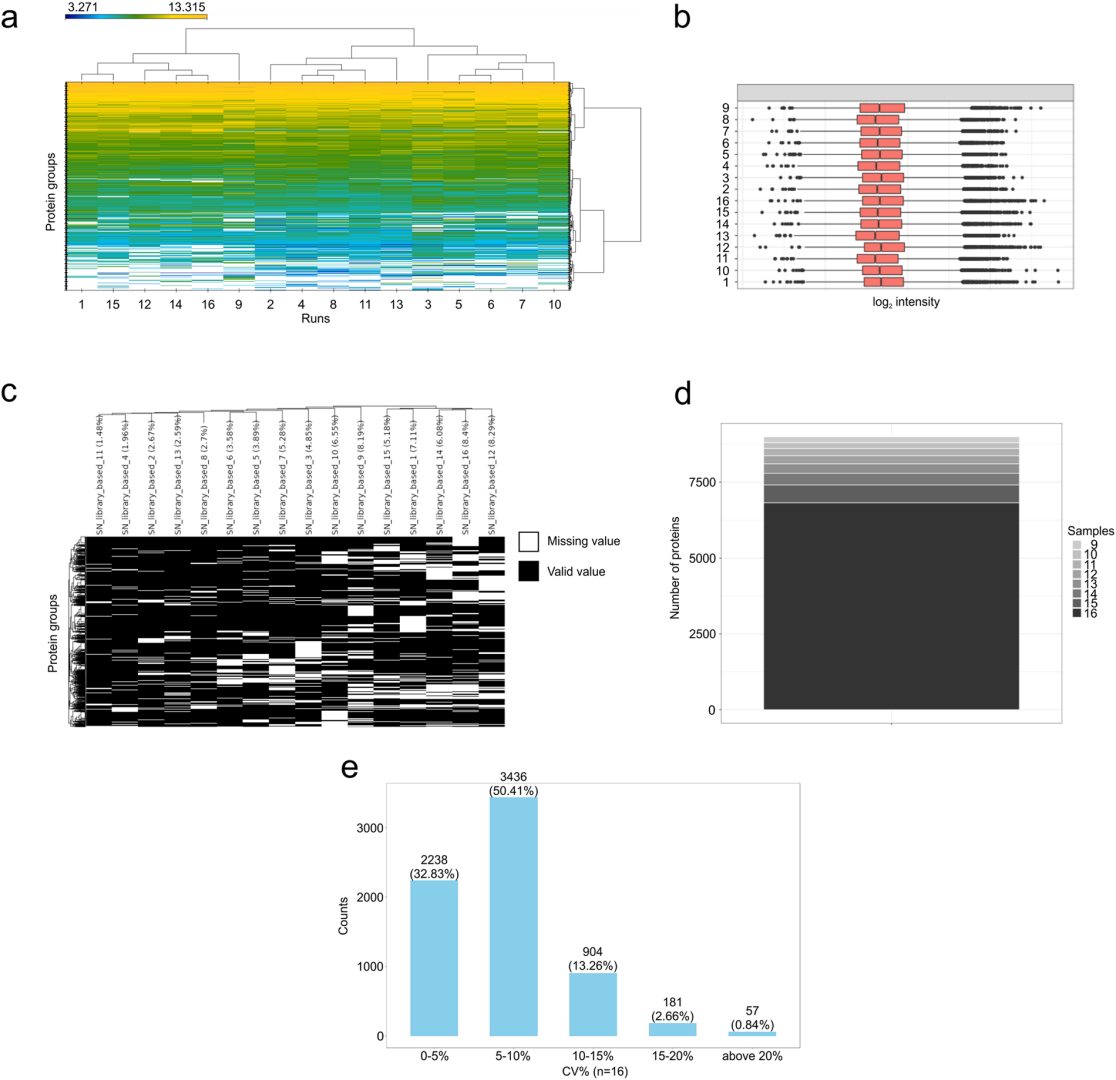
The application of our TNBC library for DIA data extraction from DIA-PASEF measurements of the 16 individual TNBC samples in Spectronaut 18.5. (**a**) Heatmap displaying the sample clustering. (**b**) Protein group quantities within individual runs. (**c**) Distribution of missing values. Only proteins with at least one missing value are visualized. (**d**) Protein identification completeness. (**e**) Distribution of CVs on the protein group level.

From the pooled and fractionated 105 TNBC samples that were used for generation of our hybrid library in Spectronaut 16.0, we selected 16 individual samples that were measured in DIA-PASEF mode and processed in the most recent software version, Spectronaut 18.5 with the use of our TNBC library. Using this approach, we identified 190,310 precursors, 140,566 stripped peptides, and 10,463 protein groups (FDR = 0.01, Supplementary file 4). The heatmap displays sample clustering, showing three potential clusters among the 16 samples (Fig. 3a). The total intensity variability between individual runs was minimal (Fig. 3b). The missing value rate varied from 1.48% to 8.4% (Fig. 3c), and 6816 protein groups were identified in all 16 samples (Fig. 3d). 83.2% and 99.1% protein groups were quantified with CV below 10% and 20%, respectively (Fig. 3e).

To compare the performance of our library with library-free approach of DIA data analysis and to compare the utilization of another widely used DIA data processing software, DIA-NN, with the Spectronaut, we show the results for the same 16 individual samples from the outcomes of library-free analysis in Spectronaut 18.5 and both library-based and library-free analyses in DIA-NN 1.8.1 in the Supplementary File 5 (the comparison results are visualized in Supplementary Figs. S2 and S3, for detailed protein group-level data please see Supplementary File 6-9).

In conclusion, in this study we present a novel proteomics assay library derived from TNBC tissues by the timsTOF technology representing the deepest coverage of TNBC proteome to date. We also report comparison of two key DIA data extraction strategies, Spectronaut and DIA-NN, and show that implementation of our library in the quantitative analysis represents a sensitive approach outperforming library-free methods. Our TNBC-specific library offers a comprehensive source for a deep proteomics characterization of TNBC tumors, leading to identification of novel therapeutic targets and precise patient stratification.

### Supplementary information

Supplementary Figures

Supplementary File 1

Supplementary File 2

Supplementary File 3

Supplementary File 4

Supplementary File 5

Supplementary File 6

Supplementary File 7

Supplementary File 8

Supplementary File 9

## Data Availability

The data analysis methods as well as software, their versions and parameters used are described in the Methods section. No custom scripts were generated in this work.
