# Supplementary material for: A hybrid DDA/DIA-PASEF based assay library for a deep proteotyping of triple-negative breast cancer
Source: Sci Data. 2024 Jul 18;11:794. doi: 10.1038/s41597-024-03632-2 (PMC11258311; doi:10.1038/s41597-024-03632-2)
Supplement: Supplementary file 1 — Supplementary Figures [file 41597_2024_3632_MOESM1_ESM.pdf]

# A hybrid DDA/DIA-PASEF based assay library for a deep proteotyping of triple-negative breast cancer

Petr Lapcik[1], Klara Synkova[1], Lucia Janacova[1], Pavla Bouchalova[1], David Potesil[2], Rudolf Nenutil[3], and Pavel Bouchal[1]

1. Department of Biochemistry, Faculty of Science, Masaryk University, Brno, Czech Republic
2. Central European Institute of Technology, Masaryk University, Brno, Czech Republic
3. Department of Oncological Pathology, Masaryk Memorial Cancer Institute, Brno, Czech Republic
corresponding author: Pavel Bouchal (bouchal@chemi.muni.cz)

## Supplementary figures

### Table of Contents

**Fig. S1** The HILIC chromatogram of peptide fractionation for TNBC spectral library generation........... 1

**Fig. S2** Application of TNBC library for quantitative data extraction in Spectronaut 18.5 and DIA-NN 1.8.1........................................................................................................................................ 2

**Fig. S3** Evaluation of performance of the DIA data extraction from the 16 individual TNBC samples using Spectronaut 18.5 and DIA-NN 1.8.1 in library-based and library-free mode ............................... 3

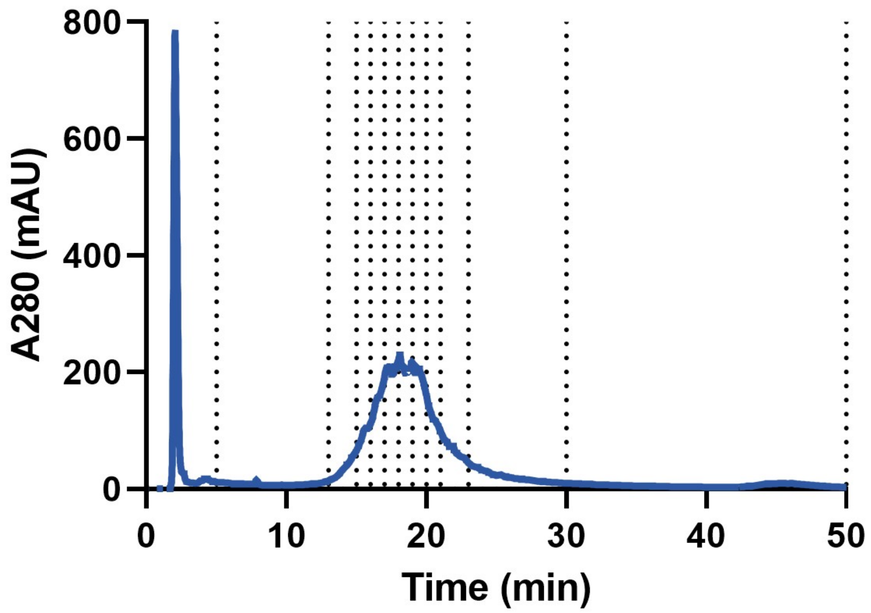


**Fig. S1** The HILIC chromatogram of peptide fractionation for TNBC spectral library generation. Peptides were fractionated to 50 fractions that were mixed into 12 final fractions as follows: fraction 1 (0-5 min); fraction 2 (5-13 min); fraction 3 (13-15 min); fraction 4 (15-16 min); fraction 5 (16-17 min); fraction 6 (17-18 min); fraction 7 (18-19 min); fraction 8 (19-20 min); fraction 9 (20-21 min); fraction 10 (21-23 min); fraction 11 (23-30 min); fraction 12 (30-50 min). The UV signal was monitored at 280 nm.

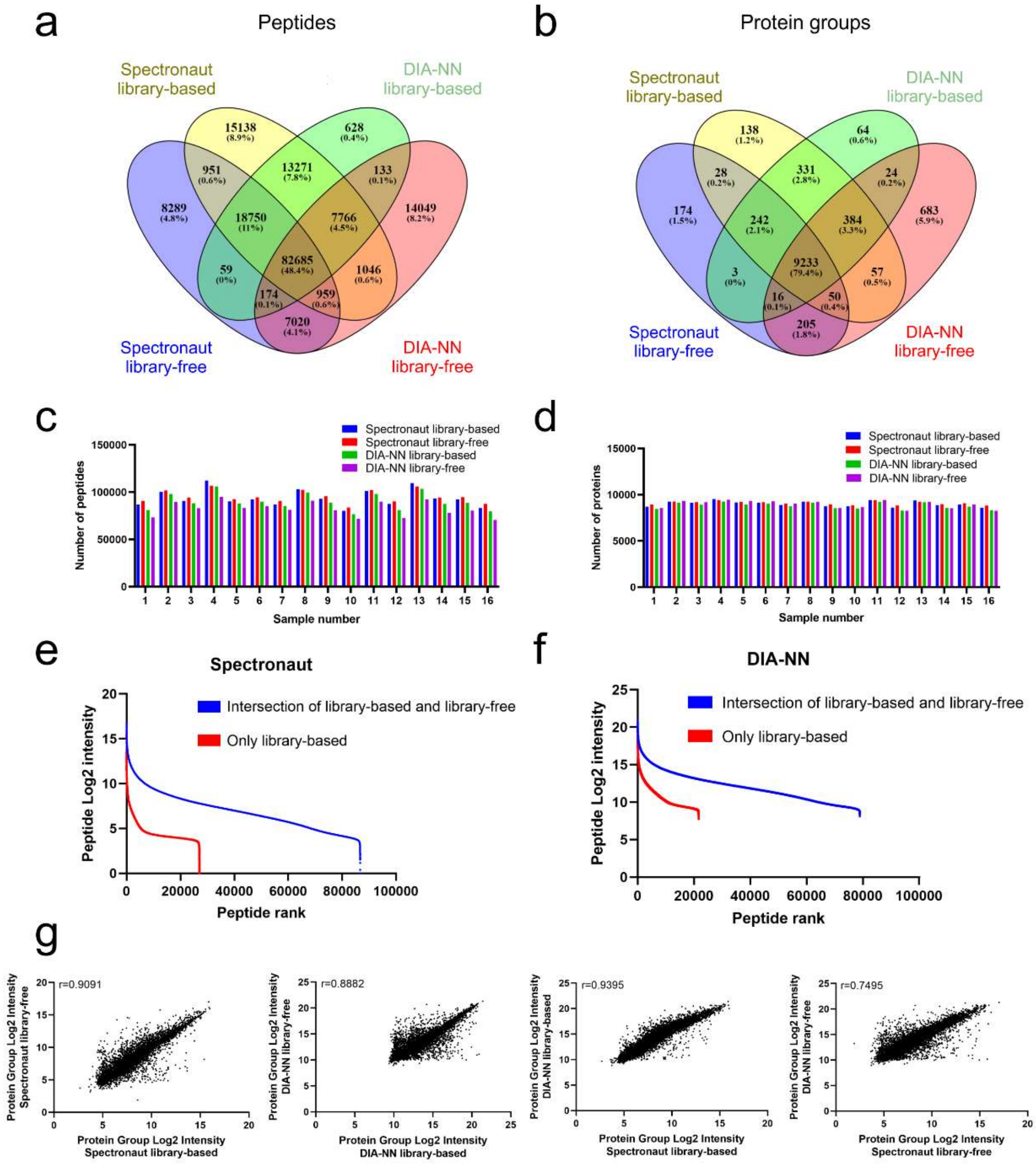


**Fig. S2** Application of TNBC library for quantitative data extraction in Spectronaut 18.5 and DIA-NN 1.8.1. Venn diagrams of identification numbers comparison between Spectronaut 18.5 and DIA-NN 1.8.1 set in library-based and library-free mode for (a) stripped peptides and (b) protein groups. Numbers of identifications in individual samples for (c) stripped peptides and (d) proteins. Intensity comparisons of peptides identified using (e) Spectronaut 18.5 and (f) DIA-NN 1.8.1 exclusively with library-based approaches and peptides identified simultaneously by both library-based and library-free methods. (g) Correlation analyses of log2 protein group intensities extracted using Spectronaut 18.5 and DIA-NN 1.8.1. Correlation coefficient r was calculated using two-tailed Pearson correlation.

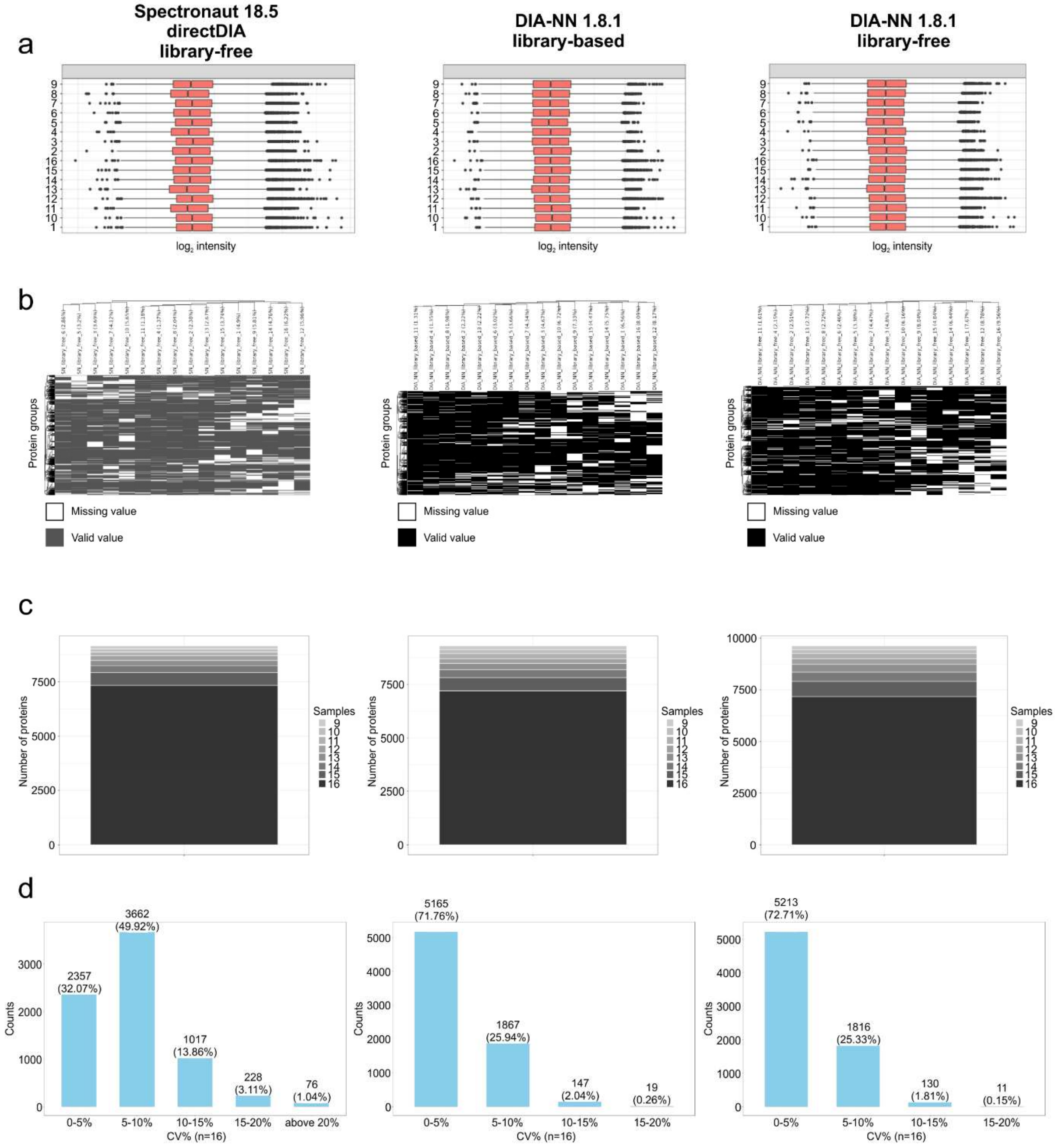


**Fig. S3** Evaluation of performance of the DIA data extraction from the 16 individual TNBC samples using Spectronaut 18.5 and DIA-NN 1.8.1 in library-based and library-free mode. (a) Protein group quantities within individual runs. (b) Distribution of missing values. Only proteins with at least one missing values are visualized. (c) Protein identification completeness. (d) Distribution of CVs on the protein group level.
