# Supplementary material for: A hybrid DDA/DIA-PASEF based assay library for a deep proteotyping of triple-negative breast cancer
Source: Sci Data. 2024 Jul 18;11:794. doi: 10.1038/s41597-024-03632-2 (PMC11258311; doi:10.1038/s41597-024-03632-2)
Supplement: Supplementary file 6 — Supplementary File 5 [file 41597_2024_3632_MOESM6_ESM.docx]

**A hybrid DDA/DIA-PASEF based assay library for a deep proteotyping of triple-negative breast cancer**

Petr Lapcik^1^, Klara Synkova^1^, Lucia Janacova^1^, Pavla Bouchalova^1^, David Potesil^2^, Rudolf Nenutil^3^, and Pavel Bouchal^1^

1. Department of Biochemistry, Faculty of Science, Masaryk University, Brno, Czech Republic

2. Central European Institute of Technology, Masaryk University, Brno, Czech Republic

3. Department of Oncological Pathology, Masaryk Memorial Cancer Institute, Brno, Czech Republic

corresponding author: Pavel Bouchal (bouchal@chemi.muni.cz)

**Supplementary Methods**

**DIA data processing in Spectronaut software using directDIA approach**

The 16 TNBC samples measured in DIA-PASEF mode used for library generation were subjected to quantitative data extraction in Spectronaut 18.5 software in library-free setting using directDIA module. Precursor Qvalue cutoff and experiment protein Qvalue cutoff were set to 0.01. No missing value imputation was used. Other parameters were set as default. The results were reported from sparse profiles including peptides identified in at least one sample with Qvalue < 0.01 ^32^.

**DIA data processing in DIA-NN software**

The 16 TNBC samples measured in DIA-PASEF mode used for quantitative data extraction in Spectronaut 18.5 were processed in DIA-NN 1.8.1 software using our hybrid library generated in Spectronaut 16.0 and in the library-free setting. For the search using our library, the Protein inference was set to Genes. Several parameters were optimized from the first experimental run and set as follows: MS1 accuracy 17 ppm, mass accuracy 12 ppm, scan window 11. Precursor FDR was set to 0.01. The unrelated runs, use isotopologues, no shared spectra, heuristic protein inference, and match between runs (MBR) were allowed. Spectral library generation was denied. Quantitative strategy was set to Robust LC (high precision). Other settings were used as default. The command “--original-mods” was added to the additional options.

For the generation of the *in silico*-predicted spectral library in DIA-NN 1.8.1 software, human UniProt/SwissProt database (version 2022_03 downloaded on 2022-09-23, 20,398 sequences) was utilized. The precursor ions were generated from in silico digest of protein database with deep learning-based spectra, retention times (RTs), and ion mobilities (IMs) prediction. The m/z range was set to 300-1800 for precursors and 200-1800 for fragment ions. The enzyme was set to trypsin/P. The fixed modification was set to methylthiolation (C), and variable modifications were set to oxidation (M) and acetylation (protein N-terminus). Precursor FDR was set to 0.01. The resulting library contains 20,373 protein isoforms and 5,757,610 precursors. The targeted data extraction of DIA-PASEF runs was performed as with the use of our library.

**Statistical analysis**

The visualizations of identification comparisons and peptide intensity comparisons were performed in Prism (GraphPad Software) version 9.5.1. Only unmodified stripped peptides were counted, the mean intensity of peptide precursors with an identical stripped sequence was used as the peptide intensity. For intensity comparison between peptides identified by library-based methods only and peptides identified simultaneously by both library-based and library-free methods, medians of each peptide intensities across 16 samples used for DIA data extraction were calculated and used for method comparisons. To compare the protein group quantification between the approaches, the two-tailed Pearson correlation was performed using Prism version 9.5.1, the medians for each protein group log2 intensities across the 16 samples were used as an input. The Venn diagrams were generated using Venny 2.1 ^37^.

**Supplementary Results**

Here we compare the outcomes of the quantitative DIA data extraction with the use of our library and with the use of the library prediction approaches in the newest versions of Spectronaut 18.5 and DIA-NN 1.8.1 software. The 16 individual samples measured in the DIA-PASEF mode were processed in the most recent software versions, Spectronaut 18.5 and DIA-NN 1.8.1 in the library-based and library-free setting. To compare outputs from these tools, we used the “qvalue sparse” setting in Spectronaut 18.5 without missing value imputation and “Protein inference” set to “Genes” in DIA-NN 1.8.1. We demonstrate that use of our library results in identification of 190,310 and 162,891 precursors, 140,566 and 123,466 stripped peptides, and 10,463 and 10,297 protein groups in Spectronaut 18.5 and DIA-NN 1.8.1, respectively (FDR = 0.01, Supplementary File 4 and 6). On the other hand, library-free setting leads to identification of 156,831 and 135,519 precursors, 118,887 and 113,832 stripped peptides, and 9,951 and 10,652 protein groups, in Spectronaut 18.5 and DIA-NN 1.8.1, respectively (FDR = 0.01, Supplementary File 7 and 8). Application of our library thus increased numbers of precursor and stripped peptide identifications by 21.4% and 18.2% in Spectronaut 18.5, respectively, and by 20.2% and 8.5% in DIA-NN 1.8.1, respectively. On the protein group level however, as the library-based setting led to increase of identifications by 5.2% in Spectronaut 18.5, the library-free approach in DIA-NN 1.8.1 reached the highest identification numbers, probably due to different protein inference strategies implemented in Spectronaut and DIA-NN. A total of 82,685 (48.4%) stripped peptides and 9,233 (79.4%) protein groups were identified using all four approaches (Supplementary Fig. S2a-b). Overall, higher identification numbers were achieved with Spectronaut 18.5 across individual samples (Supplementary Fig. S2c-d, Supplementary File 9). Peptides identified exclusively in searches against our library tended to have lower intensities compared to peptides identified simultaneously by both library-based and library-free approaches (Supplementary Fig. S2e-f), indicating library-based methods to offer superior identification of low abundant peptides. Nevertheless, the quantification across the four methods was consistent as the Pearson’s coefficients from correlation analyses of protein group intensities reported from both software tools in library-based and library-free setting was above 0.7 (Supplementary Fig. S2g).

Regarding the performance of different data processing approaches, the protein profile variability was minimal in all cases (Supplementary Fig. S3a). The observed highest rate of missing values in a single sample was 9.56% in the DIA-NN 1.8.1 dataset in library-free mode (Supplementary Fig. S3b). More than 7000 proteins were identified in all 16 samples using library-based method in DIA-NN 1.8.1 as well as with the library-free approaches in Spectronaut 18.5. and DIA-NN 1.8.1 (Supplementary Fig. S3c). Generally, the protein group CVs were lower in DIA-NN 1.8.1 outputs, as 71.76% and 72.71% protein groups had CV below 5% in DIA-NN 1.8.1 library-based and library-free output, respectively, whereas in Spectronaut 18.5 outputs of library-based and library-free methods, 32.83% and 32.07% of protein groups reached CV below 5%, respectively, and 50.41% and 49.92% had CV 5-10% (Fig. 3, Supplementary Fig. S3d). This indicates that Spectronaut 18.5 reaches more identification numbers, however DIA-NN 1.8.1 offers better reproducibility.

In summary, our results demonstrate that the use of our comprehensive TNBC-specific library outperforms the predicted libraries in the newest versions of Spectronaut 18.5 and DIA-NN 1.8.1, as it offers improved sensitivity for identification of low abundant peptides. This is in accordance with other studies ^38,39^ that report the advantage of library-free methods over the use of small libraries, however highlight improved sensitivity of methods based on large libraries over the use of library-free methods.

We moreover reached higher identification numbers in Spectronaut 18.5 compared to DIA-NN 1.8.1. software in the library-based setting. From other studies that also compared Spectronaut and DIA-NN software performance, Demichev et al. ^24^ demonstrated DIA-NN 1.6.0 software to reach higher numbers of identified precursors from experimental spectral library compared to Spectronaut 11. Moreover, in other study ^26^ DIA-NN 1.8.1 achieved identification of more precursors and proteins using the library-free approaches compared to Spectronaut 14.4 from DIA-PASEF data. In contrary, other authors ^40^ found Spectronaut 14.10 to quantify more proteins than DIA-NN (v02/04/2020) in experimental library-based setup in E. coli lysates. Lou et al ^32^ observed Spectronaut 16.1 to achieve more protein identifications for library-dependent timsTOF experiments compared to DIA-NN 1.8.1, nevertheless DIA-NN displayed superior quantification accuracy and precision, which could be associated with different peak selection and scoring strategies. However, in our study we used newer software versions than in the previous studies, and our results thus demonstrate increase in identification numbers in the newest Spectronaut software version.

**Additional references**

37. Oliveros, J.C. Venny. An interactive tool for comparing lists with Venn’s diagrams https://bioinfogp.cnb.csic.es/tools/venny/index.html (2007-2015).

38. Zhang, F. *et al.* A Comparative Analysis of Data Analysis Tools for Data-Independent Acquisition Mass Spectrometry. *Mol Cell Proteomics* **22**, 100623 (2023).

39. Wen, C. *et al.* Investigation of Effects of the Spectral Library on Analysis of diaPASEF Data. *J Proteome Res* **21**, 507–518 (2022).

40. Gotti, C. *et al.* Extensive and Accurate Benchmarking of DIA Acquisition Methods and Software Tools Using a Complex Proteomic Standard. *J Proteome Res* **20**, 4801–4814 (2021).
